# Reply to Peigneur et al. The Helix Ring Peptide U11 from the Venom of the Ant, *Tetramorium bicarinatum*, Acts as a Putative Pore-Forming Toxin, Not a New Kv1.3 Channel Blocker. Comment on “Boy et al. A New Kv1.3 Channel Blocker from the Venom of the Ant *Tetramorium bicarinatum*. *Toxins* 2025, *17*, 379”

**DOI:** 10.3390/toxins18010052

**Published:** 2026-01-19

**Authors:** Guillaume Boy, Laurence Jouvensal, Nathan Téné, Jean-Luc Carayon, Elsa Bonnafé, Françoise Paquet, Michel Treilhou, Karine Loth, Arnaud Billet

**Affiliations:** 1 Equipe BTSB EA-7417, Institut National Universitaire Champollion, Place de Verdun, 81012 Albi, France; guillaume.boy@univ-jfc.fr (G.B.); nathan.tene@univ-jfc.fr (N.T.); jean-luc.carayon@univ-jfc.fr (J.-L.C.); elsa.bonnafe@univ-jfc.fr (E.B.); michel.treilhou@univ-jfc.fr (M.T.); 2 Centre de Biophysique Moléculaire, Centre National de la Recherche Scientifique (CNRS), Unité Propre de Recherche (UPR) 4301, 45071 Orléans, France; laurence.jouvensal@cnrs-orleans.fr (L.J.); francoise.paquet@cnrs-orleans.fr (F.P.); karine.loth@cnrs-orleans.fr (K.L.); 3 Unité de Formation et de Recherche (UFR) Sciences et Techniques, Université d’Orléans, 45071 Orléans, France

We thank Peigneur et al. [1], for their interest in our recent publication entitled, “A new Kv1.3 channel blocker from the venom of the ant *Tetramorium bicarinatum*.”. In response to their Comment, we address the authors’ concerns grouped across four main themes: 1. Oxidation State of Tb11a, 2. Relevance of hKv1.3 as a Model Target, 3. Kv1.3 Blocking Potency and 4. Dyad Geometry.

## 1. Oxidation State of Tb11a

Peigneur et al. argue that their negative findings on Kv1.3 currents cannot be attributed to a reduced peptide, since their supplier certified the oxidized form. However, in their article [2], they wrote in part 2.1. U11 Peptide Synthesis and Analytical Validation: “Using a high-resolution timsTOF Flex instrument in the MALDI positive mode (Bruker, Billerica, MA, USA), Figure 1D illustrates a monoisotopic mass of 4021.1532, expressed as [M + H]^+^, indicating that the peptide we have used in our functional tests corresponds with oxidized U11 (thus with the disulfide bridge formed)”. This statement is not in accordance with what they now state in the comment: “Figure 1 shows the mass profile of Tb11a with 4019.14 as [M + H]^+^, exactly the same mass expected for the theorical mass, confirming the oxidized state of the peptide we have used.”. In our article [3], we demonstrated distinct mass differences between the oxidized and reduced peptide forms using MALDI-MS, and we provided the mass chromatogram of both peptides used in the study.

In our recent article, we provided direct experimental evidence that the reduced form exhibited no Kv1.3 blocking activity and instead induced cytotoxicity in mammalian cells, the phenotype reported by Peigneur et al. in Xenopus oocytes [2]. In their comment, Peigneur et al. noted that only HEK cells were used to test the cytotoxicity of reduced Tb11a, while a variety of human cell types (including HEK cells) were used for testing the oxidized form. The primary aim of the original study was to assess the oxidized peptide, which is why cytotoxicity assays were performed on multiple cell types with the fully formed peptide. Subsequently, LDH and CCK8 assays were conducted on HEK cells with the reduced peptide to directly investigate the toxicity hypothesis, as Peigneur et al. proposed a potential cytotoxic effect without conducting experimental tests themselves.

Thus, their results are not incompatible with ours but rather complement them by confirming the cytotoxic potential of the reduced conformer.

## 2. Relevance of hKv1.3 as a Model Target

The comment questions the use of hKv1.3 as a target to substantiate Tb11a role as an insect neuroactive peptide. Barassé et al. first described Tb11a as a neuroactive helix ring peptide in insects and hypothesized a link to potassium conductance [4].

Kv1.3 provides a structurally and physiologically relevant channel model with well-characterized pharmacology, allowing us to test this hypothesis under controlled conditions. The lack of high-quality structural data on insect Kv channels necessitates the use of human Kv orthologs as substitutes. By choosing hKv1.3, we were able to suggest by docking experiments that Tb11a engages the pore vestibule via a Lys–Tyr dyad, a canonical feature of many venom-derived Kv blockers. This validates the peptide as a K^+^ channel modulator, even if insecticidal activity involves additional targets.

## 3. Kv1.3 Blocking Potency

It is correct that Tb11a exhibits micromolar rather than picomolar potency on hKv1.3, whereas classical venom-derived Kv1.3 blockers (e.g., ShK, HsTx1) reach picomolarIC50 values. We emphasize, however, that Tb11a represents a novel structural scaffold, with a helix ring stabilized by only one disulfide bridge, distinct from the canonical disulfide-rich scaffolds evolutionarily optimized in sea anemones and scorpions for example. As highlighted in this paper, this first description of Kv1.3 blockade by a helix ring peptide should be interpreted as a proof-of-principle. The micromolar affinity therefore does not disqualify Tb11a as “promising”; rather, it broadens the structural space of Kv1.3 ligands. Potency studies by dose response, mutagenesis or chemical modification are a well-established path for pharmacological molecules development.

## 4. Dyad Geometry

The comment refers to the classical dyad distance of 6.6 ± 1.0 Å between the lysine and aromatic residues described by Dauplais et al. [5]. In our study, we performed homology modeling and docking that positioned the K25–Y21 pair of Tb11a at ~7 Å apart, directly engaging Kv1.3 pore residues in a geometry consistent with the dyad paradigm [3]. In addition, the Ta11a model presented in our paper corresponds to a predicted structure rather than an experimentally determined one. We cannot determine how much it deviates from the actual conformation of the peptide, particularly when it comes to the orientation of the side chains. We also emphasize in the text that this model does not accurately reproduce the expected distance for the dyad and should therefore not be considered as a perfect representation. Moreover, molecular structures are inherently dynamic and can adapt upon interaction with their partners. This is precisely why the docking was performed in a fully flexible manner on the peptide.
